# The Diagnostic Quandary of Primary Renal Ewing's Sarcoma on ^18^ F-FDG PET/CT Scan: A Case Report with a Thorough Review of Literature

**DOI:** 10.1055/s-0045-1810114

**Published:** 2025-07-21

**Authors:** Anuradha Pandit, Shaivy Malik, Charanjeet Ahluwalia

**Affiliations:** 1 Department of Pathology, Vardhman Mahavir Medical College and Safdarjung Hospital, New Delhi, India

**Keywords:** ^18^ F-FDG PET/CT scan, Ewing's sarcoma, kidney, renal neoplasms, small round cell sarcoma

## Abstract

Ewing's sarcoma (EWS) of the kidney is an extremely rare and aggressive malignancy, accounting for less than 1% of renal tumors. This report presents a 42-year-old male who initially presented with hematuria and intermittent fever, with imaging studies suggesting a renal cell carcinoma (RCC). However, histopathological and immunohistochemical analysis, including CD99 positivity and NKX2.2 nuclear expression, confirmed the diagnosis of primary renal EWS. The patient was promptly started on neoadjuvant chemotherapy (VAC + IE [vincristine, adriamycin, cyclophosphamide, ifosfamide, and etoposide]), which he tolerated well without significant adverse effects. Given its nonspecific clinical and ^18^ F-fluorodeoxyglucose positron emission tomography/computed tomography scan presentation, the case highlights the diagnostic challenges in distinguishing renal EWS from more common renal neoplasms such as RCC. Early diagnosis through histopathology and immunohistochemistry is crucial for guiding appropriate management. Given the aggressive nature of renal EWS, a multimodal treatment approach involving chemotherapy followed by radical nephrectomy is essential for improving prognosis. This case sheds light on the importance of considering renal EWS in the differential diagnosis of renal masses and emphasizes the role of early intervention in enhancing survival outcomes.

## Introduction

The Ewing's sarcoma family of tumors encompasses a broad spectrum of small round cell neoplasms, including osseous and extraosseous Ewing sarcoma (EWS), soft tissue primitive neuroectodermal tumors, and malignant small-cell tumors of the thoracopulmonary region, collectively known as Askin's tumor. 1 This aggressive and highly malignant group of small round cell tumors develops from mesenchymal stem cells. EWS was first characterized by James Ewing in 1921 as an embryonal round cell neoplasm exhibiting variable neuroectodermal differentiation. 2

EWS typically occurs in children and young adults with a definite preponderance for diaphysis of long bones or presents as a soft tissue round cell sarcoma. Its occurrence in adults is seldom seen in clinical practice. 3 Moreover, primary EWS of the kidneys is an infrequent clinicopathological occurrence comprising less than 1% of all renal tumors. 4 Furthermore, diagnosing renal EWS can be challenging clinically and radiologically, as it can mimic other renal neoplasms, characteristically known to occur in the kidneys.

EWS is known to exhibit an aggressive clinical course marked by rapid growth and early metastatic dissemination, most commonly to the lungs, bones, and lymph nodes, leading to a dismal prognosis. 5 A comprehensive meta-analysis estimates the 5-year disease-free survival rate for EWS to range between 45 and 55%. 6 Therefore, prompt accurate diagnosis of renal EWS remains crucial as it can aid in the early initiation of chemotherapy and help improve the chances of survival and prognostic outcome for the patient.

Herein, we present an interesting clinical tale of a 42-year-old adult male diagnosed with primary renal EWS. It provides valuable insights into the nonspecific clinical presentation in the present case study, which led to the false initial impression of renal cell carcinoma (RCC). However, a timely histopathological diagnosis on a core-needle biopsy helped clinch the diagnosis and subsequently led to the formation of correct management plans.

## Case Presentation

A 42-year-old male presented to the outpatient department with complaints of hematuria and intermittent fever (101°F) for 15 days. The patient was diagnosed with hypertension with no history of diabetes mellitus. Additionally, there was no family history suggestive of any malignancies. A general physical examination revealed no significant abnormalities. Laboratory investigations demonstrated normal urea levels, mildly elevated serum creatinine (1.5 mg/dL), and a complete blood count within physiological limits. Notably, the erythrocyte sedimentation rate was significantly elevated, whereas lactate dehydrogenase levels were within standard limits. Three consecutive days of urine cytology were negative for high-grade urothelial carcinoma.

**Fig. 1:**
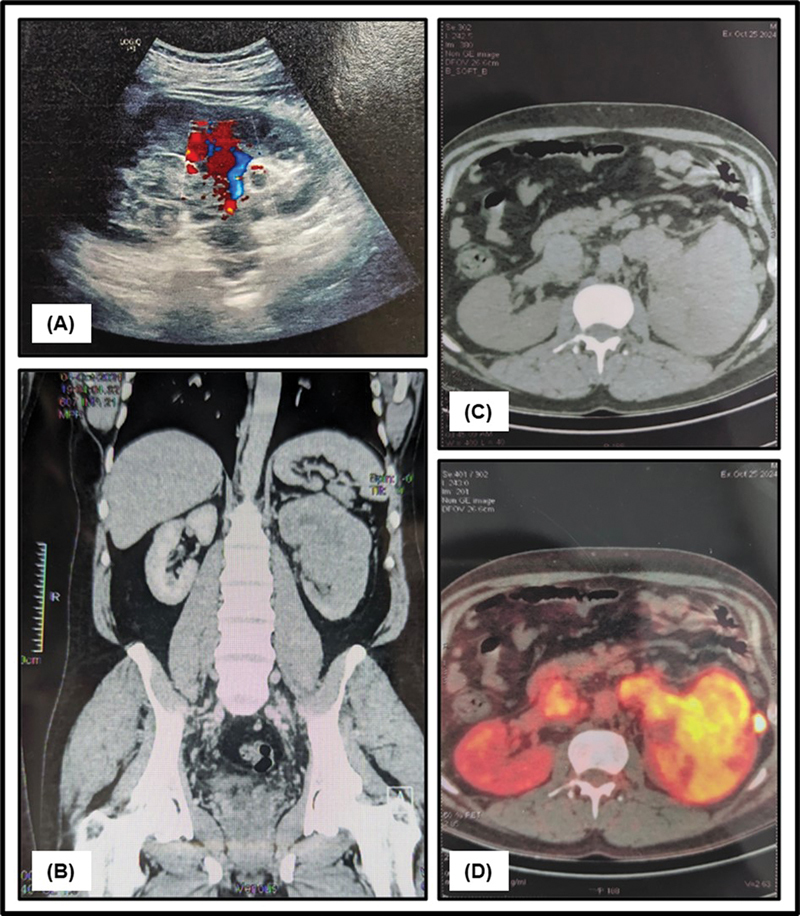
Radiological findings: ( **A** ) Color Doppler ultrasound image of the left kidney demonstrating an ill-defined renal mass with prominent internal vascularity. ( **B** ) Coronal and ( **C** ) transverse contrast-enhanced computed tomography (CT) images of the left kidney demonstrating a hypodense lesion in the upper and mid-pole of the left kidney, extending into the left renal vein and inferior vena cava (IVC) with partial thrombus. ( **D** ) Positron emission tomography/CT (PET-CT) image showing a metabolically active, large, irregular lobulated heterogeneous mass in the upper and interpolar cortex of the left kidney.

Ultrasound (USG) imaging revealed a poorly defined, heterogeneous mass measuring 10 × 9.3 cm in the upper and mid-poles of the left kidney. The lesion involved the pelvicalyceal system and pelvis, while the reniform contour of the kidney was preserved. Evidence of extension into the left renal vein and inferior vena cava (IVC) was noted. A provisional diagnosis of an RCC of the left kidney was proposed ( Fig. 1A ).

Contrast-enhanced computed tomography (CECT) of the abdomen demonstrated a hypodense lesion of approximately 10.5 × 9 × 7.8 cm in size, encasing the main and polar branches of the left renal artery and extending into the left renal vein and IVC with partial thrombus formation ( Fig. 1B and C ). Based on the USG and CECT findings, a provisional diagnosis of RCC was made.

CECT of the chest revealed a well-defined hypodense nodule in the left apical segment, likely indicative of metastatic disease. Urine cytological examination revealed no evidence of atypical cells and was negative for malignancy. A subsequent whole-body ^18^ F-fluorodeoxyglucose positron emission tomography-CT scan ( ^18^ F-FDG PET/CT scan) identified a FDG-avid, metabolically active, large, irregular, lobulated heterogeneous soft tissue density mass lesion measuring 11.1 × 9.4 × 10.1 cm (anteroposteriorly × transversally × craniocaudally) noted in the upper pole and interpolar cortex of the left kidney. Perilesional perinephric fat stranding was appreciated as well. The lesion was seen infiltrating into the left pelvicalyceal system. The left renal vein and IVC appeared distended with FDG-avid long-segment large hypodense filling defect extending to infrahepatic IVC (maximum thickness measuring 3 cm). The lesion was closely abutting the distal body of the pancreas, spleen, and lateral conal fascia. FDG-avid left perinephric nodules were noted, the largest measuring 16 × 14 mm. The maximum standardized uptake value (SUVmax) of the primary lesion was 18.8 and the SUVmax measurement was obtained via automated volume of interest method. A few FDG-avid left para-aortic and aortocaval lymph nodes were also noted, the largest measuring 3.2 × 2.4 cm, SUVmax 7.3 in the aortocaval region, suggesting lymphatic metastasis. Additionally, metabolically inactive thrombi were also detected in the bilateral common iliac veins. Metabolically active, well-defined subpleural nodules were noted in the anterior segment of the left lung upper lobe, likely metastatic. The ^18^ F-FDG PET/CT scan features were consistent with a primary RCC with involvement of the left renal vein, IVC, left para-aortic and aortocaval lymph nodes, and distant metastatic spread to the lungs as described ( Fig. 1D ).

**Fig. 2:**
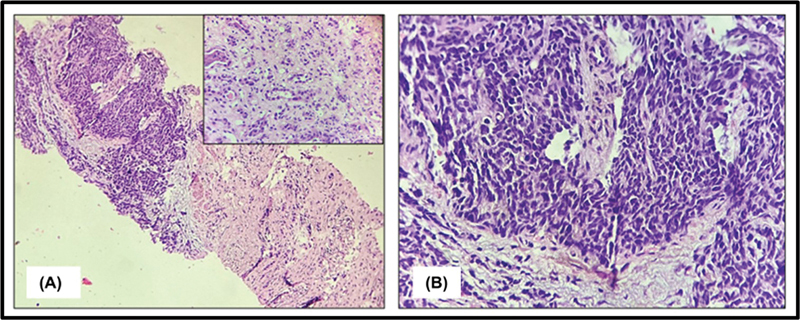
Pathological findings: ( **A** ) Hematoxylin and eosin-stained section (10 × ) demonstrating diffuse sheets of small round tumor cells infiltrating the renal parenchyma. The inset demonstrates residual normal renal tubules in the core biopsy (40 × ). ( **B** ) At 40× magnification, the tumor cells exhibit a high nuclear-to-cytoplasmic ratio, scant eosinophilic cytoplasm, hyperchromatic nuclei with irregular contours, and inconspicuous nucleoli.

A core-needle biopsy for the renal mass was performed, and the linear cores were received in the pathology department in 10% neutral-buffered formalin. They were routinely processed, and 5-µm thick serial sections were stained with hematoxylin and eosin. The histopathological examination of multiple cores revealed diffuse sheets of small round tumor cells infiltrating the renal parenchyma. These cells exhibited a high nuclear-to-cytoplasmic ratio, a scant rim of eosinophilic cytoplasm, hyperchromatic nuclei with dense chromatin, irregular nuclear membranes, and inconspicuous nucleoli. Foci of tumor necrosis were noted, and atypical mitoses were abundant, with a mitotic count of 8 to 10/ 10 high-power fields. Blood vessels within the tumor properly showed hyalinization. Histomorphological features were consistent with a small round blue cell tumor of the kidney ( Fig. 2A and B ).

**Fig. 3:**
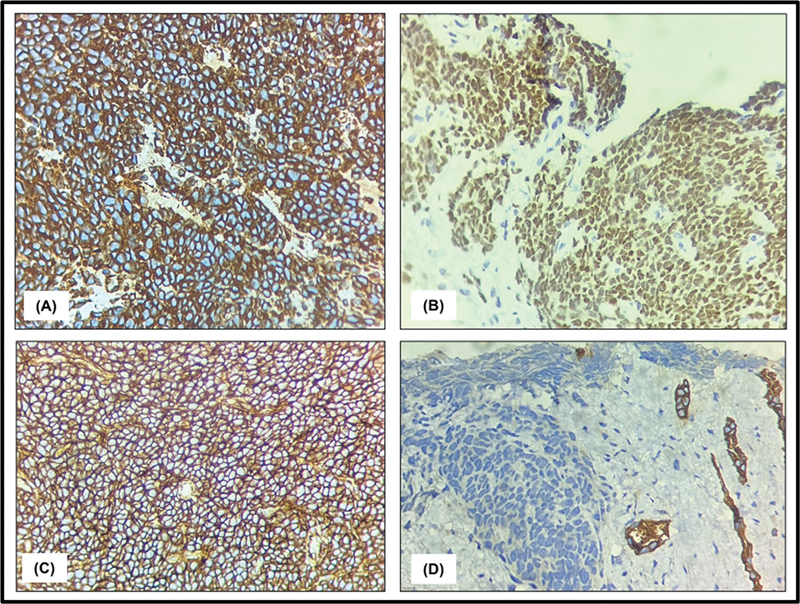
Immunohistochemistry findings: Immunohistochemical analysis revealed ( **A** ) cytoplasmic positivity for vimentin; ( **B** ) strong, diffuse nuclear expression of NKX2.2; ( **C** ) uniform diffuse membranous staining for CD99; and ( **D** ) negative staining for CK-7.

An extensive panel of immunohistochemical antibodies was applied on 2-µm-thick sections on poly-L-lysine-coated slides for exact characterization of the neoplasm. The neoplastic cells demonstrated immunopositivity for vimentin (cytoplasmic expression), CD99 (diffuse membranous positivity), NKX2.2(strong diffuse nuclear expression) ( Fig. 3 ), and ERG, and a negative immunoexpression for synaptophysin, chromogranin, pan-cytokeratin (AE1/AE3), FLI1, EMA, CK7, CK20, GATA3, LCA, CD10, WT1, MyoD1, myogenin, CD45, NSE, CD56, and PSA. K _i_ -67 proliferation index was high, around 90%. The diagnosis of lymphoma was excluded by a negative CD45 immunostaining, whereas the absence of synaptophysin, chromogranin, and CD56 expression conclusively eliminated small-cell carcinoma. Furthermore, the possibility of embryonal rhabdomyosarcoma was negated by negative immunostaining for myogenin, MyoD1, and desmin. Notably, differentials such as Wilms tumor, neuroblastoma, desmoplastic small round cell tumor, and alveolar rhabdomyosarcoma are not typically seen in this age group. The diagnosis of Wilms tumor was excluded through WT1 expression analysis, while neuroblastoma was effectively ruled out by synaptophysin and chromogranin studies. Additionally, negative staining for myogenin and MyoD1 excluded alveolar rhabdomyosarcoma, while the lack of WT1 (C-terminus) expression led to the exclusion of a desmoplastic small round cell tumor. Based on the corroborative histomorphological and ancillary findings of immunohistochemistry (IHC), a final confirmatory diagnosis of primary EWS of the left kidney with evidence of distant metastasis to the lung was rendered.

VAC + IE (vincristine, adriamycin, cyclophosphamide, ifosfamide, and etoposide) combination neoadjuvant chemotherapy was immediately initiated under strict clinical monitoring. The patient has been planned for six cycles of VAC + IE before a radical nephrectomy can be undertaken. He has been regularly receiving his designated chemotherapy cycles and is tolerating the drugs well. The patient has not shown any adverse clinical events 4 weeks into the therapy.

## Discussion

Primary EWS of the kidney is an exceedingly rare clinicopathological entity, accounting for less than 1% of renal tumors. Initially described by Seemayer et al in 1975, more than 150 cases have been documented in the literature. 4 According to Risi et al's meta-analysis of 116 cases, the median age of occurrence is 28 years, while Ellinger et al reported a mean age of 26 years across 52 cases, with an age range of 4 to 66 years. 7 8 Renal EWS predominantly affects individuals aged 20 to 30 years and exhibits a slight male predominance. 4

The clinical presentation of renal EWS is nonspecific and may include flank pain, hematuria, and/or a palpable mass. 9 Radiologically, these tumors frequently mimic RCC, thereby posing diagnostic dilemmas to clinicians and radiologists, complicating a definite diagnosis. Renal EWS typically appears as a large, poorly defined, heterogeneous mass with areas of necrosis and hemorrhage. 10 Tumor thrombus, commonly associated with clear cell RCC, occurs in only 50% of renal EWS cases. 11 While imaging modalities such as CECT provide essential clues, definitive diagnosis relies on a combination of histopathology, IHC, and genetic studies, such as fluorescence in situ hybridization. 12

Histopathologically, EWS is characterized by monomorphic diffuse sheets of small round malignant cells, often forming Homer Wright rosettes. 13 The differential diagnosis includes other small round cell tumors, such as Wilms tumor, neuroblastoma, synovial sarcoma, malignant lymphoma, alveolar rhabdomyosarcoma, small cell neuroendocrine carcinoma, desmoplastic small round cell tumor, and small cell carcinoma. 12 A comprehensive IHC panel is essential for differentiation, with EWS cells typically expressing diffuse membranous CD99, cytoplasmic vimentin, and diffuse and intense nuclear expression of NKX2.2. NKX2.2 is a novel, sensitive, and specific IHC marker for EWS and, in combination with CD 99, has proved to be highly effective in clinching the diagnosis of EWS. 14 Tumor cells typically demonstrate t(11;22)(q24;q12) *EWSR1::FLI1* (Ewing sarcoma breakpoint region 1/Friend leukemia integration 1) translocation in 85 to 90% of the cases, a hallmark of EWS. 15 However, in the remaining meager fraction of the cases, *EWSR1::ERG* translocation can be present, as was evident in the present case by negative FLI1 immunoexpression and positive ERG immunoexpression. Therefore, diagnosing primary renal EWS necessitates a multifaceted diagnostic approach involving histopathological examination, ancillary techniques of IHC, and molecular analysis.

There is no established consensus for managing renal EWS owing to its rare occurrence. However, a multimodal approach combining surgery, chemotherapy, and radiotherapy is recommended. Chemotherapeutic regimens often include vincristine, dactinomycin, cyclophosphamide, doxorubicin, ifosfamide, and etoposide. 9 Emerging targeted therapies, such as insulin-like growth factor-1 receptor antibodies, have shown promise in addressing aggressive disease patterns. 16 However, treatment outcomes remain grim, with the median survival rate for metastatic disease hovering at 15 months. 17 For localized disease, early nephrectomy combined with chemotherapy has demonstrated a 4-year survival rate of up to 85%, emphasizing the importance of early intervention. 1 Advancements in molecular and immunological profiling have opened avenues for novel treatments. Immunotherapy and molecularly targeted drugs may offer new hope for managing refractory or metastatic cases. Further research into optimizing adjuvant and neoadjuvant therapies is crucial to improving patient outcomes. Radiotherapy is particularly valuable for cases with incomplete resection or when surgical margins are positive. It is also used for palliation in metastatic settings. 9

Table 1 describes the complete gamut of clinicopathological findings of previously described cases of primary renal EWS.

**Table 1:** Clinicopathological spectrum of previously reported cases of primary renal Ewing's sarcoma

Study	Year	Age/ sex	Symptoms	Imaging	Histopathology	IHC	Molecular findings (FISH)	Treatment
Khudair et al 15	2024	38/F	Abdominal pain and two episodes of nonbilious vomiting. Six months of progressive abdominal distension and intermittent constipation	CECT: 25 × 18 × 18 cm heterogeneous noncalcific left renal exophytic mass. Retroperitoneal adenopathies and multiple bilateral pulmonary nodules were also noted	Small round blue cell tumor with a densely sclerosed stroma and marked necrosis	Positive: CD99 and FLI-1. Focal weak positivity: synaptophysin and an intact INI-1. Negative: Desmin, myogenin, pancytokeratin, and WT-1	Positive: EWSR1 translocation (11:22)	VDC protocol and sodium 2-mercaptoethane sulfonate, followed by IE
Senkhum et al 11	2023	22/M	Gross hematuria for 3 weeks	Multiphase CT: 14.4 cm × 14.3 cm × 9.7 cm heterogeneous enhancing mass at the right kidney extended along the right hepatic vein, intrahepatic and hepatic IVC without intra-abdominal metastasis	Ewing's sarcoma	Positive: CD99	Not reported	Neoadjuvant chemotherapy with four cycles of doxorubicin and ifosfamide, followed by open radical nephrectomy and tumor thrombus removal
İlhan et al 12	2023	54/M	Right flank pain and hematuria	The heterogeneously enhanced hyperdense cystic mass lesion, ∼74*63 mm in size, is located in the upper pole of the right kidney	Nuclear pleomorphism, spindle cells, and rosette formation	Positive: vimentin, CD99, FLI-1 Negative: Synaptophysin, SMA, myogenin, and CK18	Positive: 30% of tumor cells show EWSR1 rearrangement	Right radical nephrectomy, adjuvant chemotherapy regimens alternately every 3 weeks
Singh et al 5	2023	48/F	Difficulty in passing urine for 5 months associated with pain in the right flank region, with an episode of hematuria 5 months back	CECT abdomen: A large, lobulated, heterogeneously enhancing retroperitoneal mass lesion that had completely replaced the right kidney PET/CT: 14.8 × 18.7 × 20.6 cm	Small round cell neoplasm	Positive: NFX-2.2, FLI-1, CD-99, synaptophysin. K _i_ 67 proliferation index: 70 80%	Not reported	The patient succumbed to the disease before treatment could be initiated
Choudhury et al 19	2022	18/F	Left-sided flank pain and hematuria after minor trauma to the left flank	CECT abdomen: A large hypodense, heterogeneously enhancing mass in the upper pole of the left kidney	Multiple small round blue cells with a high nucleo-cytoplasmic ratio	Positive: CD99 **Negative:** LCA, WT-1, CD-33, CD-20, chromogranin, and synaptophysin	Not reported	Combination chemotherapy comprising of cyclophosphamide, etoposide, and mesna
Cheng et al 20	2020	31/F	Intermittent pain in the left flank and a palpable abdominal mass	Enhanced CT: A hypoechoic mass of 18 cm × 14.5 cm × 14 cm from the left kidney with central necrosis, no invasion to renal veins or inferior vena cava	Small round cell tumor with focal necrosis	Positive: AE1/AE3, CD99, CD56, synaptophysin (Syn), K _i_ -67 (50%) Negative: S-100, EMA, CgA, WT1, desmin, SMA, and MyoD1	Positive: t(22q12), EWS-FLI1 type 1 translocation	Open, left radical nephrectomy followed by adjuvant chemotherapy
Alghamdi et al 21	2019	15/F	Left flank pain and abdominal distention with weight loss	CT abdomen: a mass of 22*20*25 cm on the left kidney, significantly impinging on adjacent structures	Small round cell tumor with extensive necrosis	Positive: CD99 (12E7) and showed retained INI-1 expression	Positive: EWSR1 (22q12) rearrangement	Exploratory open laparotomy and left radical nephrectomy followed by combination chemotherapy comprising vincristine, doxorubicin, and cyclophosphamide (VDC) alternating with ifosfamide/etoposide (IE)
Zhang et al 4	2019	18/M	Persistent hematuria and left lower lumbar discomfort for 6 months	7.3 cm × 7.0 cm × 9.0 cm heterogeneous lobulated mass invading the mid-part of the left kidney with areas of necrosis and hemorrhage, accompanied by parenchyma invasion and expansion of the left kidney and signs of main renal vein invasion	Monotonous population of small round cells with extensive necrosis	Positive: CD99, FLI-1, and synaptophysin	Not reported	Laparoscopic nephrectomy followed by adjuvant chemotherapy and local radiotherapy
Sadiq et al 9	2017	14/F	Pain in the right flank	CT: 8.8 × 7.7 cm ^2^ heterogeneous mass involving the mid- and lower pole of the right kidney with a large necrotic component and invading the perinephric fat and ipsilateral vein	Small round blue cell neoplasm	Strongly positive: CD99 and FLI1 Patchy positive: synaptophysin and CKAE1/AE3. Negative: TLE and WT-1	Not reported	Right nephrectomy followed by multiagent chemotherapy
Choubey et al 13	2017	17/F	Right flank pain for 2 weeks	CECT: heterogeneously enhancing 12 × 11 cm mass occupying the interpolar region and lower pole of the right kidney	Small round cell neoplasm	Positive: vimentin and CD99 Negative: cytokeratin, WT1, chromogranin, and myogenin	Not reported	Right radical nephrectomy with right hemicolectomy. Adjuvant etoposide-based chemotherapy
Almeida et al 22	2014	19/M	Sudden sharp right flank pain, accompanied by low-grade fevers and vomiting	Infiltrative mass in the upper pole of the right kidney, invading the right liver	Ewing sarcoma/primitive neuroectodermal tumor	FISH: Translocations involving the EWS locus ( *EWSR1* Gene rearrangement)	Positive: EWSR1 rearrangement	Multiagent chemotherapy
Kairouani et al 17	2012	40/F	Severe back pain 1 month after a right nephrectomy was performed because of a renal mass	190 mm × 100 mm × 90 mm mass	A malignant tumor composed of monomorphic cells. These cells fitted together in some places, forming rosettes	Positive: CD99 and focally positive for vimentin anticorps and protein S-100 Negative: Epithelial membrane antigen (EMA), leucocyte common antigen (LCA), and desmin.	Not reported	Six cycles of chemotherapy every 3 weeks
Present study	2024	42/M	Hematuria and intermittent fever (101°F) for 15 days	CECT abdomen: A hypodense lesion ∼9 × 7.8 cm in size, encasing the main and polar branches of the left renal artery and extending into the left renal vein and IVC with partial thrombus formation	Small round cell neoplasm	Positive: vimentin, NKX-2.12, CD-99, and ERG Negative: FLI1, synaptophysin, chromogranin, pan-cytokeratin (AE1/AE3), EMA, CK7, CK20, GATA3, LCA, CD 10, WT1, MyoD1, myogenin, CD45, NSE, CD56, and PSA K _i_ 67 proliferation index: 90%	−	6 cycles of VAC + IE (vincristine, adriamycin, cyclophosphamide, ifosfamide, and etoposide) combination neoadjuvant chemotherapy before radical nephrectomy

Abbreviations: CECT, contrast-enhanced computed tomography; CT, computed tomography; FISH, fluorescence in situ hybridization; F, female; IE, ifosfamide and etoposide; IHC, immunohistochemistry; IVC, inferior vena cava; M, male; PET, positron emission tomography; VDC, vincristine, doxorubicin, and cyclophosphamide.

Recent studies have provided valuable insights into prognostic factors. Factors such as localized disease, absence of thrombus, and responsiveness to chemotherapy significantly improve survival. Conversely, local extension, tumor thrombus in the renal vein or IVC, and distant metastasis correlate with worse outcomes. PET/CT imaging plays a pivotal role in staging, treatment monitoring, and assessing residual disease posttherapy. 5 Renal EWS is highly aggressive, with early metastatic spread and a poor prognosis. Common metastatic sites include the lungs, liver, lymph nodes, and bones, with over 65% of patients presenting with distant metastases. 5 15

Despite its invaluable utility in staging and detecting metastatic disease, ^18^ F-FDG PET/CT imaging has notable limitations, particularly in differentiating among histologically diverse renal tumors. In the present case, the intense FDG avidity of the renal mass (SUVmax 18.8), perinephric nodules, and pulmonary lesions initially raised suspicion for metastatic RCC. However, this interpretation underscores a critical pitfall: FDG uptake reflects metabolic activity rather than tumor type and is therefore not specific to RCC. It can be elevated in other high-grade malignancies such as EWS due to their high metabolic activity. Moreover, the presence of tumor thrombus in the renal vein and IVC, a feature classically associated with RCC, further confounded the imaging impression. These findings highlight the inherent limitation of relying solely on metabolic imaging for tumor characterization. In rare and atypical presentations, such as primary renal EWS, definitive diagnosis requires histopathological confirmation supported by immunohistochemical and molecular profiling.

While PET/CT serves as a valuable tool for staging and monitoring therapeutic response, it remains insufficient as a standalone modality for definitive tumor characterization. However, emerging PET tracers, including those targeting fibroblast activation protein (FAPI) and somatostatin receptors, show promise in enhancing the diagnostic specificity for various sarcoma subtypes. Among these, 68Ga-FAPI PET/CT has demonstrated higher uptake in sarcomas compared with ^18^ F-FDG, particularly in low-grade tumors where FDG uptake is often low and nonspecific. This suggests that 68Ga-FAPI may provide improved tumor visualization and diagnostic precision in sarcoma imaging, especially in cases where FDG PET/CT is suboptimal. 18 This case underscores the importance of maintaining a broad differential when interpreting PET/CT results, especially in patients with uncommon age demographics or atypical tumor biology.

Collaborative studies are essential for establishing evidence-based management protocols for this rare but devastating malignancy.

## Conclusion

This case study sheds light on the importance of considering primary renal EWS in the plausible differentials of renal neoplastic mass lesions despite their exceedingly rare occurrence and atypical presentations. It also highlights the pivotal role of histopathological evaluation and ancillary techniques in establishing a definite diagnosis. Furthermore, prompt initiation of neoadjuvant chemotherapy remains vital in improving patient prognosis in cases of renal EWS, which is otherwise notorious for exhibiting an aggressive clinical course with grim outcomes.
